# Combining the absorptive and radiative loss in metasurfaces for multi-spectral shaping of the electromagnetic scattering

**DOI:** 10.1038/srep21462

**Published:** 2016-02-19

**Authors:** Wenbo Pan, Cheng Huang, Mingbo Pu, Xiaoliang Ma, Jianhua Cui, Bo Zhao, Xiangang Luo

**Affiliations:** 1 State Key Laboratory of Optical Technologies on Nano-Fabrication and Micro-Engineering, Institute of Optics and Electronics, Chinese Academy of Science, P. O. Box 350, Chengdu 610209, China

## Abstract

The absorptive and radiative losses are two fundamental aspects of the electromagnetic responses, which are widely occurring in many different systems such as waveguides, solar cells, and antennas. Here we proposed a metasurface to realize the control of the absorptive and radiative loss and to reduce the radar cross section (RCS) in multi-frequency bands. The anti-phase gradient and absorptive metasurfaces were designed that consists of metallic square patch and square loop structure inserted with resistors, acting as an phase gradient material in the X and Ku band, while behaving as an absorber in the S band. The simulation and experiment results verified the double-band, wideband and polarization-independent RCS reduction by the absorptive and anti-phase gradient metasurfaces.

The control of the electromagnetic wave have attracted increasing attention along with the growing up of the metamaterials area, which is devoted to design and control the electromagnetic waves in exotic manner which could not be achieved using traditional (natural) materials^1^,^2^,^3^,^4^,^5^,^6^,^7^,^8^,^9^,^10^,^11^,^12^,^13^,^14^,^15^,^16^,^17^,^18^. One big prospect of the metamaterials is that they could lead to the invisibility cloaking of objects, which has been demonstrated in both the microwave and optical frequencies^1^,^2^,^3^. Nevertheless, metamaterial cloaks have suffered from many drawbacks such as limited size and extremely narrow bandwidth. Alternatively, scientists have realized that metamaterials could be designed in other ways to realize functionalities similar to the cloaks. As has been demonstrated in many stealth aircrafts, it was widely known that the radar scattering signal could be controlled by changing the shape or covering radar absorbing materials on the surface. In essence, these methods can be considered as the manipulation of the loss of the scattered radar signals. On the one hand, the radar absorbing material could convert the incident radar wave into heat or other kind of energy, thus the reflecting signal could be reduced^4^,^5^,^6^,^7^,^8^,^9^,^10^,^11^,^12^,^13^,^14^,^15^. On the other hand, using shaped geometries, the reflected wave could be guided to other directions that do not point to the radar receivers, thus the radar cross section (RCS) could also be dramatically reduced^19^,^20^,^21^,^22^. The later loss of scattering signal could be considered as one kind of radiative loss of optical resonant systems^22^,^23^,^24^.

Recently, two-dimensional metamaterials (metasurfaces) have shown their unique capacity in the control of both the absorption loss and radiation properties^25^,^26^,^27^,^28^,^29^,^30^,^31^,^32^,^33^,^34^,^35^. Many metasurface-based absorbers have been demonstrated and caused much interest, due to its low mass and low profile. However, because of the resonance property of the metamaterial, the absorption is typically restricted at a narrow band. A great variety of methods for increasing the absorbing band have been proposed, such as multi-resonance elements and multi-layer structures^30^,^31^. Besides the perfect metamaterial absorber, phase-gradient metasurfaces could be also used to shape the electromagnetic shape of the objects with nearly no influence on the geometric properties^32^,^33^,^34^,^35^. By arranging the meta-atoms in a space/size/shape-variant manner, the reflected phases could be arbitrarily controlled, so that the backscatter waves could be steered to pre-designed directions. Therefore, the scattered electromagnetic field of metallic or dielectric targets is changed, and thus the real target shape cannot be identified.

Traditionally, the radar absorbing and phase-gradient metasurfaces were thought to be independent of each other. Here we proposed, for the first time, a metasurface that could control the absorption and phase gradient simultaneously. Through constructing different element period in the lower band and higher band, the metasurface would have different absorption and scattering performance. Within the higher frequency band, this metasurfaces can be realized by combining different element structures with anti-phase, and near full reflection performance is desired. In the lower frequency band, most of the incoming wave would be absorbed by the metasurface and converted into heat energy. Both simulated and measured results have fully verified the high-efficiency RCS reduction at S and X-Ku bands.

## Results

**Figure 1:**
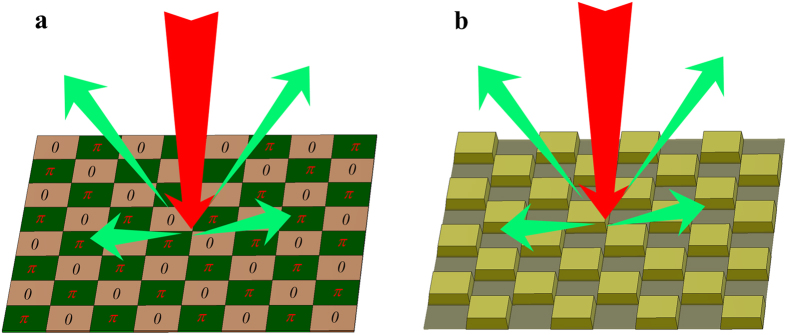
Principle of the virtual shaping based on anti-phase metasurface. (**a**) Schematic of the scattering from a planar surface with anti-phase metasurface under normal illumination. (**b**) The scattered waves from a metallic surface with different step heights under normal illumination.

The main objective of this paper is to design a combination of absorptive and phase gradient metasurfaces that can achieve RCS reduction at two broad bands. Figure 1 shows the schematic of phase gradient metasurface. It is composed of two different elements with 180° reflection phase difference in a chessboard-like configuration. In this alternating circumstance, the virtual shape of the metasurface is just like a chessboard pattern with different step heights, as shown in Fig. 1(b). When the incoming wave is incident on this metasurface, the reflection wave along the normal would be cancelled out and the backscatter energy is redirected to other angles. Therefore, the phase gradient metasurface is able to reduce the RCS by utilizing the anti-phase reflection property to alter the scattering patterns.

An *m *× *n* planar array with element spacing of *d* is adopted to quantitatively investigate the RCS reduction performance of the phase gradient metasurface. *m* and *n* represent the number of the array element in the horizontal and vertical directions, respectively. The scattered phase of each element is assumed to be *ϕ (m, n)*, and all the elements are assumed to have the equal reflection magnitude. When a plane wave is perpendicularly incident onto this surface, the predicted far-field pattern (the array factor) can be expressed as^35^:


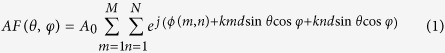


where *θ* and *φ* are the elevation and azimuth angles of an arbitrary direction, respectively. Compared to a same-size PEC surface, the RCS reduction of the metasurface can be calculated as^33^,^34^:


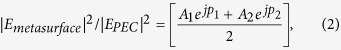


where *E*_*metasurface*_ and *E*_*PEC*_ are the total reflection field of the metasurface and PEC surface, respectively. *A*_*1*_ and *A*_*2*_ are the reflection magnitudes of the two elements of metasurface, and *P*_*1*_ and *P*_*2*_ are their corresponding reflection phases. Since each kind of elements is seized of half area of the entire metasurface^34^, by applying (2), a 10 dB RCS reduction can be realized when the phase difference (|*P*_*1*_–*P*_*2*_|) is within 180° ± 37°.

**Figure 2:**
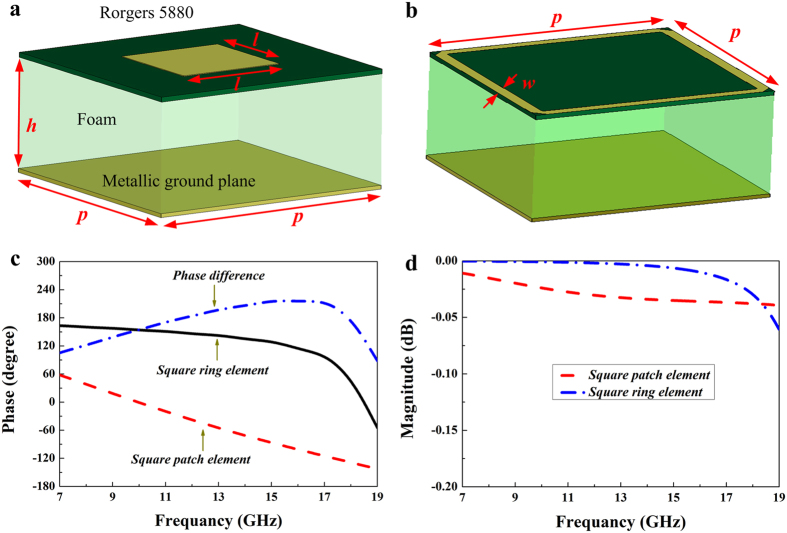
Two cell geometry of the phase gradient metasurface and their simulated reflection characteristics. (**a**) Square patch. (**b**) Square ring. (**c**) The reflection phases of the square patch and square ring structures and their phase difference. (**d**) The reflection magnitude of the square patch and square ring structures. The geometrical parameters of the two elements are optimized as follows: *l *= 1.7 mm, *h *= 6.5 mm, *p *= 4.15 mm, and *w *= 0.2 mm.

**Figure 3:**
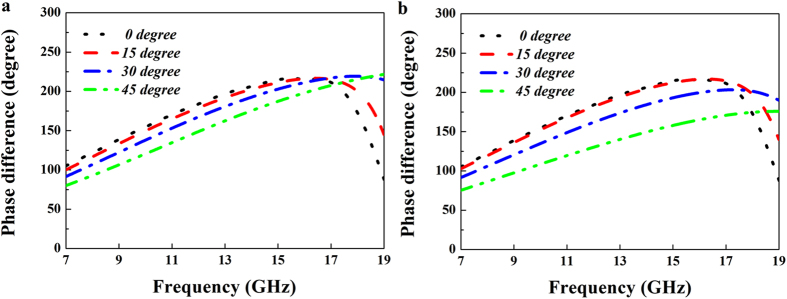
Reflection phase difference between the square patch and square ring elements for different incident angles. (**a**) TE polarization. (**b**) TM polarization.

Figure 2 shows the two different elements of the phase gradient metasurface. One element is a metallic square patch (with dimensions of *l × l*) etched on one side of Rogers 5880 substrate (*ε *= 2.2) with a thickness of 0.127 mm. On the other side of the substrate is a foam spacer (*ε*_*r*_ = 1.1 and *h *= 6.5 mm) backed by a metallic ground plane. The other element is a metallic square ring, as seen in Fig. 2(b). The width of the metallic square ring is assumed to be *w*. In order to simplify optimization of the structure parameters, both two types of elements have the same period of *p*. Numerical simulation is carried out to investigate their reflection performance by using commercial software CST Microwave Studio 2014. To obtain the S-parameters of each element, the infinite periodic array model is adopted. Through parameter sweep, reflection phase difference of these two elements is optimized between 143° and 217° in X and Ku bands. Figure 2(c) depicts the reflection phases of these two elements and the phase difference between them. It is seen that reflection phase difference value is close to 180° (± 37°) in a wide frequency band ranging from 9.3 to 18.3 GHz. The reflection magnitudes are plotted in Fig. 2(d), and it shows that these two elements can achieve almost full-reflection and their reflection losses are both less than 0.05 dB. To investigate the frequency response of reflection phase for the designed two elements under the oblique incidence, we analyze the reflection phase difference between them for the different incident angles of the TE and TM incident wave, as shown in Fig. 3. It is seen that the bandwidth in which the reflection phase difference is between 143° and 217° is obviously decreased with the increase of the oblique incident angle. When the incident angle varies from 0° to 45°, the starting frequency for the phase difference of 143° is shifted towards a higher frequency, and between 17 GHz and 19 GHz, the phase difference variation is very strong. As a whole, both two elements can still achieve the required reflection phase difference at a broad band for the ± 45° oblique incidence at two polarization modes.

**Figure 4:**
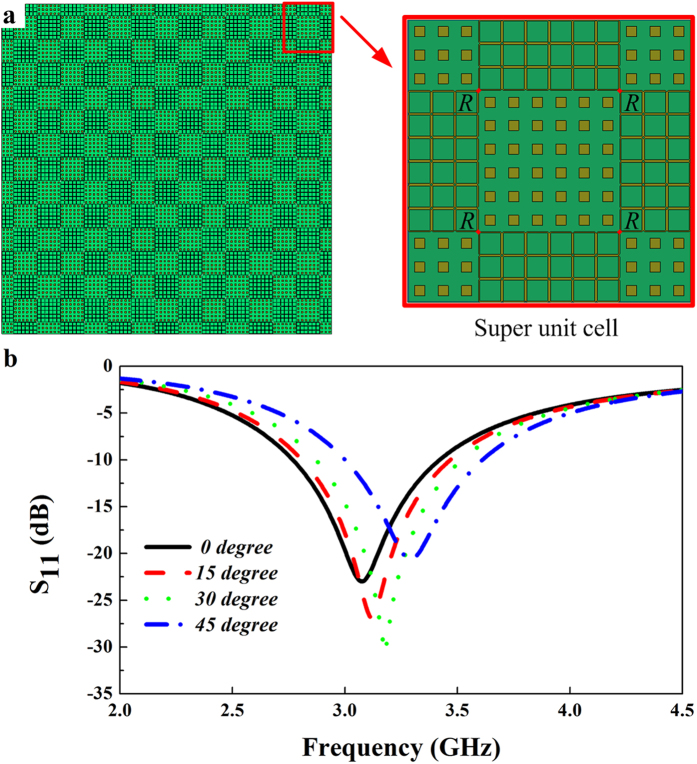
Super unit cell for the absorptive metasurface and its simulated reflection characteristic. (**a**) Geometry of the absorbing element. (**b**) Reflection magnitude for different incident angles.

In order to verify RCS reduction property, the above two types of elements are distributed in a chessboard-like configuration to construct the phase gradient metasurface. Next, we employ some resistors in the designed metasurface to achieve absorption in S band, and their introduction should be required to have no influence on the anti-phase scattered fields produced by the phase gradient metasurface at X and Ku bands. Here the dimension of the chessboard (the number of two types of elements) is very important, since it not only determines the absorptive frequency, but also influences the RCS reduction performance in the higher frequency band. The absorptive performance mainly depends on the resistance value of the inserted resistors. Through optimizing these two parameters, the desired result can be obtained. Figure 4(a) shows the super unit cell of the whole metasurface that contains nine tiles. The central tile consists of 6* *× 6 square patch elements. The four tiles at each corner are composed of 3* *× 3 square patch elements, and the rest of four tiles contain 3* *× 6 square ring elements. Four chip resistors (resistance value is 820 Ω) are employed into the four gaps between the neighboring tiles for square ring elements. When this super unit cell is illuminated by the normal EM incident wave, there is little reflection in the frequency band from 2.65 GHz to 3.65 GHz. The reflection coefficient is less than −7 dB (absorption is over 80%), and it is less than − 10 dB from 2.75 to 3.43 GHz, as seen in Fig. 4(b). When the incident angle of the TE wave varies from 0° to 45°, the minimum reflection position is continuously shifted from 3.08 GHz to 3.32 GHz, but the relative bandwidths for the reflection coefficient ≤ −10 dB are all over 20% for the four different incident angles. Therefore, the designed metasurface is expected to achieve the RCS reduction at S, X and Ku band, and it is also effective for the large incident angle.

**Figure 5:**
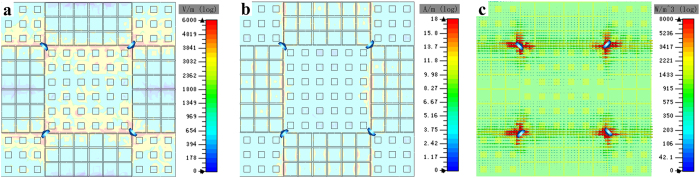
Simulated field distribution of absorbing element under normal incidence at 3.1 GHz. (**a**) Electric field distribution. (**b**) Magnetic field distribution. (**c**) Power loss density distribution.

The distribution of electric field, magnetic field and power loss density under the normal incidence at 3.1 GHz are investigated to gain an intuitive understanding of the absorption resonances. As Fig. 5(a) shows, the electric fields mainly assemble around the four chip resistors. That means the maximum charge accumulates at the corners between the neighboring tiles for square ring elements. The charge characteristics are different at the neighboring tiles, which produces capacitance. The distribution of the magnetic field is mainly around the both left and right sides of the tiles for square ring elements, as seen in Fig. 5(b). Therefore, the inductance is produced by these continuous square rings. The power loss density distribution is shown in Fig. 5 (c). It is obvious that most of the power loss is located around the chip resistors. According to the *RLC* equivalent circuit theory, we can deduce that the designed metasurface behaves strong resonance absorption at this frequency band, and most of the incoming wave is restricted to the chip resistors as Ohmic loss.

**Figure 6:**
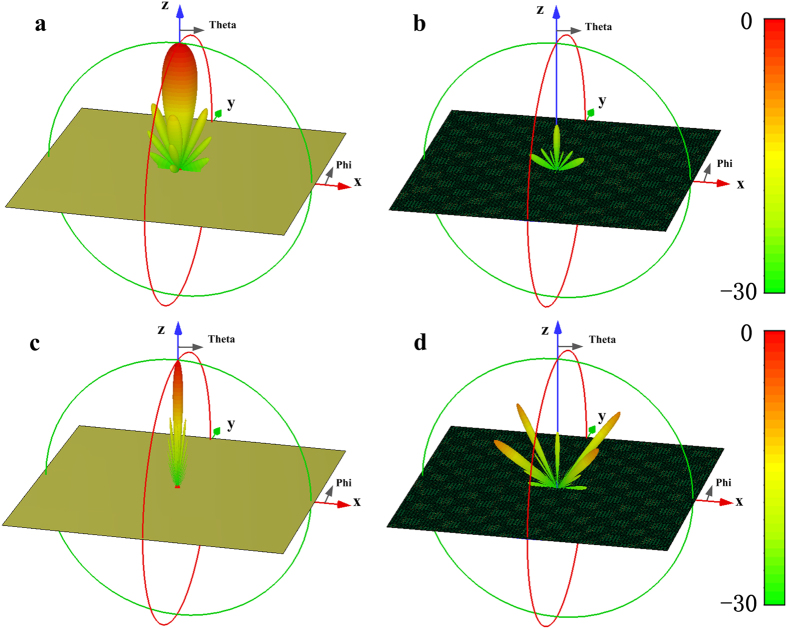
Full-wave simulation results of 3D bistatic RCS patters under normal incidence of EM waves. (**a**,**b**) The flat PEC surface and the metasurface at 3.3 GHz. (**c**,**d**) The flat PEC surface and metasurface at 11.5 GHz.

**Figure 7:**
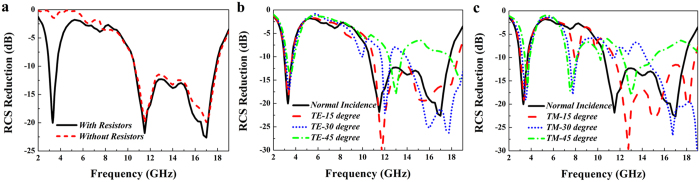
Simulated RCS reduction of the designed metasurface versus frequency from 2 GHz to 19 GHz. (**a**) The RCS reduction of the metasurface with and without resistors for normal incidence. (**b**) The RCS reduction for various incident angles with TE polarization. (**c**) The RCS reduction for various incident angles with TM polarization.

To further illustrate the absorption performance of the metasurface, the 3-D RCS of the flat PEC surface and the designed metasurface at 3.3 GHz are respectively, presented in Fig. 6(a,b). The metasurface consisting of 7* *× 7 super unit cells is designed and its overall dimension is 348.6 mm* *× 348.6 mm. Compared with the strong backscatter energy of the flat PEC surface, the RCS of the metasurface is significantly reduced, and the peak of RCS reduction exceeds 20 dB. Fig. 6(c,d) show the scattering field distribution at 11.5 GHz. The RCS is dramatically reduced by about 19 dB along the principal planes (XZ, YZ) and the main power is scattered into four directions which are *phi *= 45°, 135°, 225° and 315°. Comparing Fig. 6(b–d), the employment of two different physical mechanisms produces the different scattering field distribution. In the lower frequency, most of the incoming wave is absorbed, resulting in the obvious reduction of the backscatter energy in a broad anglewidth, while in the higher frequency, the metasurface has no absorptive property and just changes the backscatter energy direction, making the normal reflection of the incident wave sharply suppressed. The RCS reduction of the metasurface with and without resistors for the normal incidence is shown in Fig. 7(a). The metasurface without resistors shows excellent RCS reduction in 10.7–18.1 GHz, while the employed resistors in the metasurface can further make the RCS sharply reduced in 2.8–3.65 GHz. And it is still found that the RCS reduction curves of these two cases almost overlap in X and Ku band, indicating that the chip resistors have no influence on the scattering performance in the higher frequency band. Fig. 7 (b,c) show the RCS reduction characteristics for different incident angles of the TE- and TM-polarized plane waves. It is seen that the RCS reduction bandwidth is decreased as the incident angle varies from 0 to 45° due to the phase aberrations. However, the proposed metasurface still keeps low RCS property for all the cases. That means the metasurface has the large angle width to reduce RCS. If some optimization works, such as particle swarm and genetic algorithms^35^,^36^,^37^, are utilized to design this metasurface, the better result could be expected.

**Figure 8:**
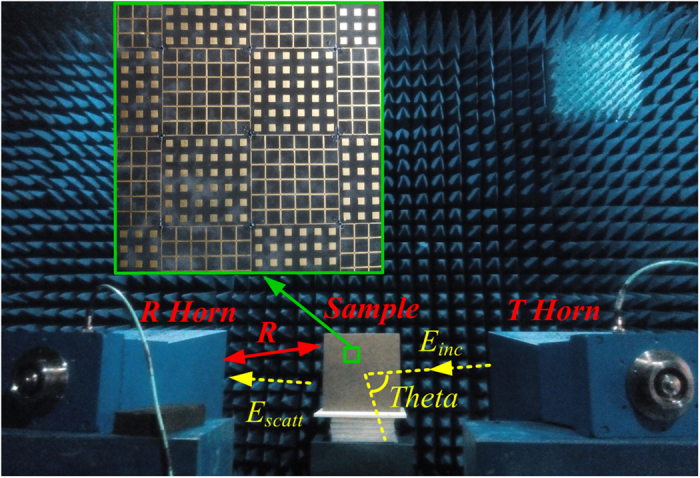
Photograph of RCS measurement setup for the designed metasurface.

**Figure 9:**
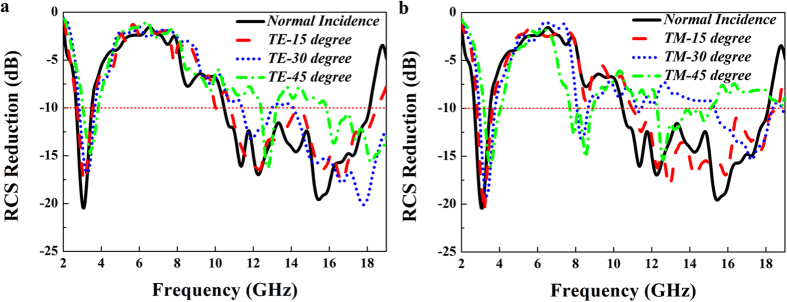
Measured bistatic RCS reduction of the designed metasurface versus frequency at different angles of incidence. (**a**) TE polarization. (**b**) TM polarization.

In order to demonstrate the performance of the designed low RCS metasurface, the metasurface composed of 7* *× 7 super unit cells has been fabricated and tested, as seen in Fig. 8. Two wideband horn antennas connected to a vector network analyzer are utilized as transmitter and receiver, respectively. Due to the absorption characteristic, the metasurface makes the incident EM wave converted into heat energy in the S band, while its anti-phase reflection property can alter the direction of the fields scattered by the metasurface. Figure 9 shows the frequency response curve of the measured RCS reduction over the frequency band of 2–19 GHz for the TE and TM polarizations incidence EM wave. For the incoming wave with normal incidence, the RCS is reduced over 10 dB in dual-band covering 2.7–3.5 GHz and 10.5–18 GHz. Thus, the RCS reduction bandwidth is calculated to be over 25% in S band and over 50% in X and Ku band. The maximum value of the RCS reduction reaches as high as 20.5 dB at 3.1 GHz, and 19.5 dB at 15.5 GHz.

With the increase of the incident angle, the RCS reduction is also considerable as expected. It is seen that the absorbing frequency is shifted towards a higher frequency and the bandwidth of RCS reduction is slightly decreased as the incident angle varies from 0° to 45° for both TE and TM modes. That means the designed metasurface has the ability in absorbing most of the incoming wave with large oblique incident angle in S band. In X and Ku band, the reflection phase difference would have obvious variation on the case of oblique incidence, which is inevitable to influence the effect of RCS reduction. As the incident angle is increased from 0° to 45°, the RCS reduction performance is gradually degraded due to the phase aberrations, but the RCS is still reduced by more than 7 dB from 10.5–18 GHz for both TE and TM wave with each incident angle. Therefore, low RCS properties are still kept for the proposed metasurface at both bands (S band and X–Ku band).

## Discussion

We have proposed the novel metasurface to achieve RCS reduction at two broad bands. Two different physical mechanisms are adopted to control the backscatter field. Both simulation and experiment results show that the designed metasurface absorbs most of the incoming wave at S band of 2.7–3.5 GHz. In X–Ku band of 10.5–18 GHz, the metasurface cancels out the reflection of the normally incident plane wave and redirects the backscatter energy to other angles, making the real target have a virtual shape. The RCS reduction performance can be further improved by optimizing the reflection cells distribution of the metasurface^35^,^36^,^37^. This new design provides a good method to achieve RCS reduction at S/X/Ku band, which is important for stealth applications in the future.

## Methods

### Numerical simulations

The square patch element, square ring element and the absorbing element of the metasurface were simulated by using commercial software CST Microwave Studios 2014, with unit cell boundary. The CST Microwave Studios is carried out to investigate the full model performance of the metasurface by adopting open (add space) boundary conditions.

### Measurement

The sample placed on the foam stack is illuminated by the incoming EM wave from the transmitting horn, and the scattering wave from the sample can be received by the receiving horn. The antennas and sample are separated by a far enough distance R = 4 m to avoid the near field effect. The performance of the proposed metasurface was measured in the anechoic chamber and compared with that of metallic flat plates of the same size and geometry. The transmitting and receiving horn antennas placed on the circumference trace, where the incidence angle is equal to the reflection angle.

## Additional Information

**How to cite this article**: Pan, W. *et al.* Combining the absorptive and radiative loss in metasurfaces for multi-spectral shaping of the electromagnetic scattering. *Sci. Rep.* **6**, 21462; doi: 10.1038/srep21462 (2016).
